# Crystal structure and kinetic analysis of the class B3 di-zinc metallo-β-lactamase LRA-12 from an Alaskan soil metagenome

**DOI:** 10.1371/journal.pone.0182043

**Published:** 2017-07-27

**Authors:** María Margarita Rodríguez, Raphaël Herman, Barbara Ghiglione, Frédéric Kerff, Gabriela D’Amico González, Fabrice Bouillenne, Moreno Galleni, Jo Handelsman, Paulette Charlier, Gabriel Gutkind, Eric Sauvage, Pablo Power

**Affiliations:** 1 Cátedra de Microbiología, Departamento de Microbiología, Inmunología y Biotecnología, Facultad de Farmacia y Bioquímica, Universidad de Buenos Aires, Buenos Aires, Argentina; 2 Consejo Nacional de Investigaciones Científicas y Técnicas (CONICET), Buenos Aires, Argentina; 3 InBioS, Centre d’Ingénierie des Protéines, Université de Liège, Liège, Belgium; 4 Department of Molecular, Cellular and Development Biology, Yale University, New Haven, CT, United States of America; Dong-A University, REPUBLIC OF KOREA

## Abstract

We analyzed the kinetic properties of the metagenomic class B3 β-lactamase LRA-12, and determined its crystallographic structure in order to compare it with prevalent metallo-β-lactamases (MBLs) associated with clinical pathogens. We showed that LRA-12 confers extended-spectrum resistance on *E*. *coli* when expressed from recombinant clones, and the MIC values for carbapenems were similar to those observed in enterobacteria expressing plasmid-borne MBLs such as VIM, IMP or NDM. This was in agreement with the strong carbapenemase activity displayed by LRA-12, similar to GOB β-lactamases. Among the chelating agents evaluated, dipicolinic acid inhibited the enzyme more strongly than EDTA, which required pre-incubation with the enzyme to achieve measurable inhibition. Structurally, LRA-12 contains the conserved main structural features of di-zinc class B β-lactamases, and presents unique structural signatures that differentiate this enzyme from others within the family: (i) two loops (α3-β7 and β11-α5) that could influence antibiotic entrance and remodeling of the active site cavity; (ii) a voluminous catalytic cavity probably responsible for the high hydrolytic efficiency of the enzyme; (iii) the absence of disulfide bridges; (iv) a unique Gln116 at metal-binding site 1; (v) a methionine residue at position 221that replaces Cys/Ser found in other B3 β-lactamases in a predominantly hydrophobic environment, likely playing a role in protein stability. The structure of LRA-12 indicates that MBLs exist in wild microbial populations in extreme environments, or environments with low anthropic impact, and under the appropriate antibiotic selective pressure could be captured and disseminated to pathogens.

## Introduction

Antimicrobial resistance is a worrisome issue in healthcare worldwide during the last two decades. This led the WHO to declare April 7^th^ 2011 as the World Health Day, stating, “no action today means no cure tomorrow”. (http://www.who.int/mediacentre/news/statements/2011/whd_20110407/en/index.html). This global commitment to develop action plans on antimicrobial resistance was reaffirmed this year at UN based on the “Global Action Plan on Antimicrobial Resistance” (http://www.who.int/mediacentre/news/releases/2016/commitment-antimicrobial-resistance/en/).

Among the frightening arsenal of resistance mechanisms we face today, β-lactamases still represent the most relevant threat. The increasing prevalence of enzymes like the carbapenemases NDM-1 and KPC [1–3], and the “pandemic” CTX-M [4, 5], or the “explosive” emergence of the carbapenem-hydrolyzing class D β-lactamases (CHDL) [6, 7], are good examples of this issue. In addition to NDM-1, other metallo-β-lactamases (MBLs) such as VIM and IMP are commonly found among clinical pathogens [8].

Metallo(zinc)-β-lactamases or class B β-lactamases (EC 3.5.2.6) belong to a protein superfamily including more than 6,000 members that share a common fold and a handful of conserved motifs. They are divided into at least 15 families primarily based on their biological functions, for which the classification does not strictly reflect their phylogenetic relationship [9, 10]. Zinc-dependent β-lactamases are clustered in Group 1 of the superfamily, and they were recently sub-divided in three sub-classes (B1, B2 and B3) based on their amino acid sequences and specific structural features [11, 12].

In general, B1 and B3 sub-classes bind two zinc ions as cofactors in their active sites, and exhibit broad spectrum activity. They have been described in several bacterial species. Sub-class B2 MBLs are mono-zinc enzymes with strong carbapenemase activity, and are inhibited upon binding of a second Zn(II), and described only in *Serratia* and *Aeromonas* [10].

Soil is considered as a very rich reservoir of antimicrobial resistance genes, and several studies have demonstrated that some prevalent β-lactamase-encoding genes were recruited from the chromosome of environmental species [13, 14] and disseminated to pathogens. The case of the CTX-M β-lactamases, derived from *Kluyvera* species, is a well-studied example [14–18]. On the other side, several MBLs produced by environmental microorganisms have been identified in the last years, constituting a threat to public health if they are successfully captured and expressed in pathogens [10, 19].

This global scenario prompted scientists to intensively study unexplored reservoirs of resistance genes besides those associated with human pathogens. Many authors reported the importance and relevance of the potentially rich armory of resistance markers, collectively known as the “resistome”, present among environmental microorganisms supposedly not exposed to highly selective concentrations of antibiotics [20, 21]. It was therefore suggested that the observed resistance among these isolates was probably be due to dissemination of resistant bacteria and exchange of resistance genes from antibiotic-exposed settings by horizontal transfer and clonal expansion of the resistant sub-populations [22].

Metagenomics has provided access to antimicrobial resistance determinants from the environmental resistome. The strength of metagenomics relies on the use of methodologies aimed at the direct recovery of DNA from uncultured microorganisms, generally involving the construction of metagenomic libraries in *Escherichia coli*, and circumventing culturing for gene discovery [23]. In addition, functional metagenomic analysis can use convenient screening methods based on selective media that enable growth of those clones producing a specific enzyme or protein [24, 25].

Antimicrobial resistance genes for various families of antibiotics from diverse environmental samples, including soil, as well as from human samples have been discovered through metagenomic analysis [26–32].

Several β-lactamase-encoding genes have been recovered from soil sampled from the Bonanza Creek Experimental Forest near Fairbanks, Alaska. They represented all four Ambler classes, with a preponderance of metallo-β-lactamases (MBLs) [33]. These β-lactamases were named LRA (for “β-lactam resistance Alaskan”). Among those found, several metallo-β-lactamases (MBL) are related to known class B β-lactamases from environmental microorganisms [33].

In this study, we analyzed the kinetic properties of LRA-12 β-lactamase, and determined the crystallographic structure of the enzyme in order to evaluate the similarities and differences with prevalent metallo-β-lactamases associated with clinical pathogens.

## Materials and methods

### Bacterial strains and plasmids

*Escherichia coli* XL1-Blue (Stratagene, USA) and *Escherichia coli* BL21(DE3) (Novagen, USA) were hosts for transformation experiments.

Recombinant construction pβLR12 is a pCC1FOS derivative (CopyControl Fosmid Library Production Kit, Epicentre, USA) harboring a >30-kb insert containing the *bla*_LRA-12_ gene [33].

Plasmid vectors pTZ57R/T vector (InsTAclone PCR Cloning kit, Thermo Scientific, USA) and kanamycin-resistant pET28a(+) (Novagen, Germany) were used for routine cloning experiments and for enzyme’s overproduction, respectively.

### Recombinant DNA methodologies

The LRA-12 encoding gene was amplified by PCR from recombinant fosmid pβLR12, using 3 U PrimeSTAR HS DNA polymerase (Takara, USA) and 1 μM LRA12-NheF2 (5’ CTTTCCCTTGCTAGCCAAAAGGT-3’) and LRA12-BamR1 (5’- CTGATGGTCAAGGATCCATTTTCC-3’) primers, containing the *Nhe*I and *Bam*HI restriction sites, respectively (underlined in the sequences), allowing the cloning of the MBL coding sequence. The PCR product was first ligated in a pTZ57R/T vector, introduced in *E*. *coli* XL1-Blue competent cells by transformation, and the insert was sequenced for verification of the identity of *bla*_LRA-12_ gene and generated restriction sites, as well as the absence of aberrant nucleotides. The resulting recombinant plasmid (pTZ-12) was then digested with *Nhe*I and *Bam*HI, and the released insert was subsequently purified and cloned in the corresponding *Nhe*I-*Bam*HI sites of a pET28a(+) vector. The ligation mixture was used to first transform *E*. *coli* XL1-Blue competent cells, and after selection of recombinant clones, a second transformation was performed in *E*. *coli* BL21(DE3) competent cells and cultured on LBA plates supplemented with 30 μg/ml kanamycin. Selected positive recombinant clones were sequenced for confirming the identity of the *bla*_LRA-12_ gene, and the recombinant clone *E*. *coli* BL-12 harboring pET28/LRA-12 plasmid was obtained for protein expression experiments.

DNA sequences were determined at Macrogen Inc. (South Korea). Nucleotide and amino acid sequence analyses were performed by NCBI (http://www.ncbi.nlm.nih.gov/) and ExPASy (http://www.expasy.org/) analysis tools.

Amino acid sequences of LRA-12 and other reference class B3 metallo-β-lactamases were retrieved from the NCBI’s protein database for multi-alignment and phylogenic analysis, using the following accession numbers: LRA-12 (ACH58990), GOB-1 (ABO21417), FEZ-1 (CAB96921), L1 (CAB75346), THIN-B (CAC33832), MBL1B (CAC48262), CAU-1 (CAC87665), BJP-1 (NP_772870), SPG-1 (WP_063864745), CPS-1 (WP_063857696), ESP-1 (WP_027382699).

### Antimicrobial susceptibility

Minimum inhibitory concentrations (MICs) of β-lactam antibiotics were determined by the broth microdilution method (BMD), following CLSI’s guidelines [34], using 96-well microtiter plates, which were incubated 18 h at 35°C.

### LRA-12 production and purification

Overnight cultures of fresh recombinant *E*. *coli* BL-12 (harboring pET28/LRA-12 plasmid construction) were diluted (1/400) in 250 ml Lysogeny Broth (LB) containing 30 μg/ml kanamycin and grown at 37°C until 0.7 OD units (λ = 600 nm). At this point, culture was supplemented with 1 mM IPTG and 0.5 mM ZnSO_4_, and cultures were grown overnight at 20°C (200 rpm stirring). After centrifugation at 8,000 rpm for 20 min (4°C) in a Sorvall RC-5C (GS3 rotor), cells were resuspended in 50 mM Tris buffer + 200 mM NaCl (pH 8.0; buffer A), and 150 U benzonase, 4 mM MgCl_2_, 5 mg/ml streptomycin, and 2 mM PMSF added.

Crude extracts were obtained by ultrasonic disruption (9 cycles of 2 min, at 0°C), and clarified by centrifugation at 10,000 rpm for 20 min (4°C, SS34 rotor). Clear supernatants containing the LRA-12 metallo-β-lactamase were dialyzed overnight against 2 L buffer A, filtrated through 0.45 μm pore-size membranes, supplemented with 10 mM imidazole (300 μl of buffer B: buffer A + 500 mM imidazole, pH 8.0), and loaded onto HisTrap HP affinity columns (GE Healthcare Life Sciences, USA), connected to an ÄKTA-purifier (GE Healthcare, Uppsala, Sweden), and equilibrated with buffer A. The column was extensively washed to remove unbound proteins, and LRA-12 β-lactamase was eluted with a linear gradient (0–100%; 1 ml/min flow rate) of buffer B.

Eluted fractions were screened for β-lactamase activity *in situ* during purification by an iodometric system using 500 μg/ml ampicillin as substrate [35], and analyzed by SDS-PAGE in 12% polyacrylamide gels. Active fractions were dialyzed against buffer A, and the histidine tag was eliminated by thrombin digestion (16 hs at 25°C), using 5U of thrombin per mg protein for complete proteolysis.

Digestion mixture was then loaded onto HiTrap SP HP columns (GE Healthcare Life Sciences, USA) equilibrated in buffer A2 (100 mM Tris, 1 mM ZnSO_4_, pH 6.5), and pure mature LRA-12 was eluted with a linear gradient (0–50%; 1 ml/min flow rate) of buffer B2 (buffer A2 + 500 mM NaCl).

Protein concentration and purity were determined by the BCA-protein quantitation assay (Pierce, Rockford, IL, US) using bovine serum albumin as standard, and by densitometry analysis on 15% SDS-PAGE gels, respectively.

### Steady-state enzyme kinetics

Steady-state kinetic parameters were determined using a T80 UV/VIS spectrophotometer (PG Instruments Ltd, UK), monitoring the hydrolysis of β-lactams substrates by purified LRA-12 by following the absorbance variation at the corresponding wavelength. Briefly, each assay was completed in 10 mM HEPES, 20 mM NaCl (pH 7.5) buffer. Reactions were performed in a total volume of 500 μl at room temperature. The steady-state kinetic parameters *K*_m_ and *V*_max_ were obtained under initial-rate as described previously [36], with non-linear least squares fit of the data (Henri Michaelis-Menten equation) using GraphPad Prism version 5.03 for Windows, (GraphPad Software, San Diego California USA, www.graphpad.com):

$$v=\frac{V_{max}\times [S]}{K_{m}+[S]}$$ (1)

For low *K*_m_ values, the *k*_cat_ values were derived by evaluating the complete hydrolysis time courses as described by De Meester *et al* [37]. For competitive inhibitors, inhibition constant *K*_*i*_ was determined by monitoring the residual activity of the enzyme in the presence of various concentrations of the drug and ceftazidime 120 μM as reporter substrate; corrected *K*_i_ (considered as the observed or apparent *K*_m_) value is finally determined using the Eq 2:

$$K_{i}=\frac{K_{i\;obs}}{\left(1+[S]\right)/K_{m(S)}}$$ (2)

where *K*_m(S)_ and [S] are the reporter substrate’s *K*_m_ and fixed concentration used, respectively.

The following extinction coefficients and wavelengths were used: nitrocefin (Δε_482_ = +15,000 M^-1^.cm^-1^), benzyl-penicillin (Δε_235_ = –775 M^-1^.cm^-1^), ampicillin (Δε_235_ = –820 M^-1^.cm^-1^), piperacillin (Δε_235_ = –820 M^-1^.cm^-1^), cephalothin (Δε_273_ = –6,300 M^-1^.cm^-1^), cefuroxime (Δε_260_ = –7,600 M^-1^.cm^-1^), cefoxitin (Δε_260_ = –6,600 M^-1^.cm^-1^), ceftazidime (Δε_260_ = –9,000 M^-1^.cm^-1^), cefotaxime (Δε_260_ = –7,500 M^-1^.cm^-1^), cefepime (Δε_260_ = –10,000 M^-1^.cm^-1^), imipenem (Δε_300_ = –9,000 M^-1^.cm^-1^), meropenem (Δε_300_ = –6,500 M^-1^.cm^-1^), ertapenem (Δε_300_ = –775 M^-1^.cm^-1^) and aztreonam (Δε_318_ = –750 M^-1^.cm^-1^).

### Inactivation by chelating agents

Inhibition of enzymatic activity by ethylenediaminetetraacetic acid (EDTA) and pyridine-2,6-dicarboxylic acid (dipicolinic acid; DPA) was assayed by measuring the hydrolysis of 170 μM ertapenem after incubating the enzyme for 20 min at 25°C in the presence of different concentrations of EDTA, following residual activity of LRA-12 in 50 mM sodium phosphate buffer (pH 7.5); for DPA, hydrolysis of ertapenem was measured immediately after the addition of the different concentrations of the chelating agent. The inhibition parameter IC_50_ was assessed as the chelating agent’s concentration able to inhibit 50% LRA-12 activity.

### Crystallization of LRA-12

Crystals were grown at 20°C by hanging drop vapor diffusion with drops containing 0.2 μL of LRA-12 solution (15 mg/ml) and 0.2 μL of 0.2 M ammonium sulfate, 0.1 M Tris pH 8.5, 25% w/v polyethylene glycol (PEG) 3350.

### Data collection, phasing, model building and refinement

Data were collected at 100 K on a Dectris (Pilatus 6M) detector at a wavelength of 0.97857 Å on Proxima 1 beamline at the Soleil Synchrotron (Saint Aubin, France). Indexing and integration were carried out using XDS [38], and the scaling of the intensity data was accomplished with XSCALE [39].

Structure modeling was achieved by molecular replacement through Phaser and Buccaneer pipeline in CCP4 suite [40], using FEZ-1 structure (PDB 1JT1) as starting model. Refinement of the model was carried out using REFMAC5 [41], and TLS [42].

### Models visualization and representations

Model visualization and representation was performed with WinCoot 0.8.2 [43], and PyMol 1.7 (www.pymol.org) [44]. Topology diagram of the secondary structures of LRA-12 was performed with PDBsum [45]. Prediction of cavities within the β-lactamase was performed with KVFinder plugin for PyMol [46].

### Protein Data Bank (PDB) accession number

The coordinates and structure factors of the native LRA-12 β-lactamase were deposited under accession code 5AEB (released: 2015-09-16).

## Results and discussion

### LRA-12 is a class B3 metallo-β-lactamase

**Fig 1:**
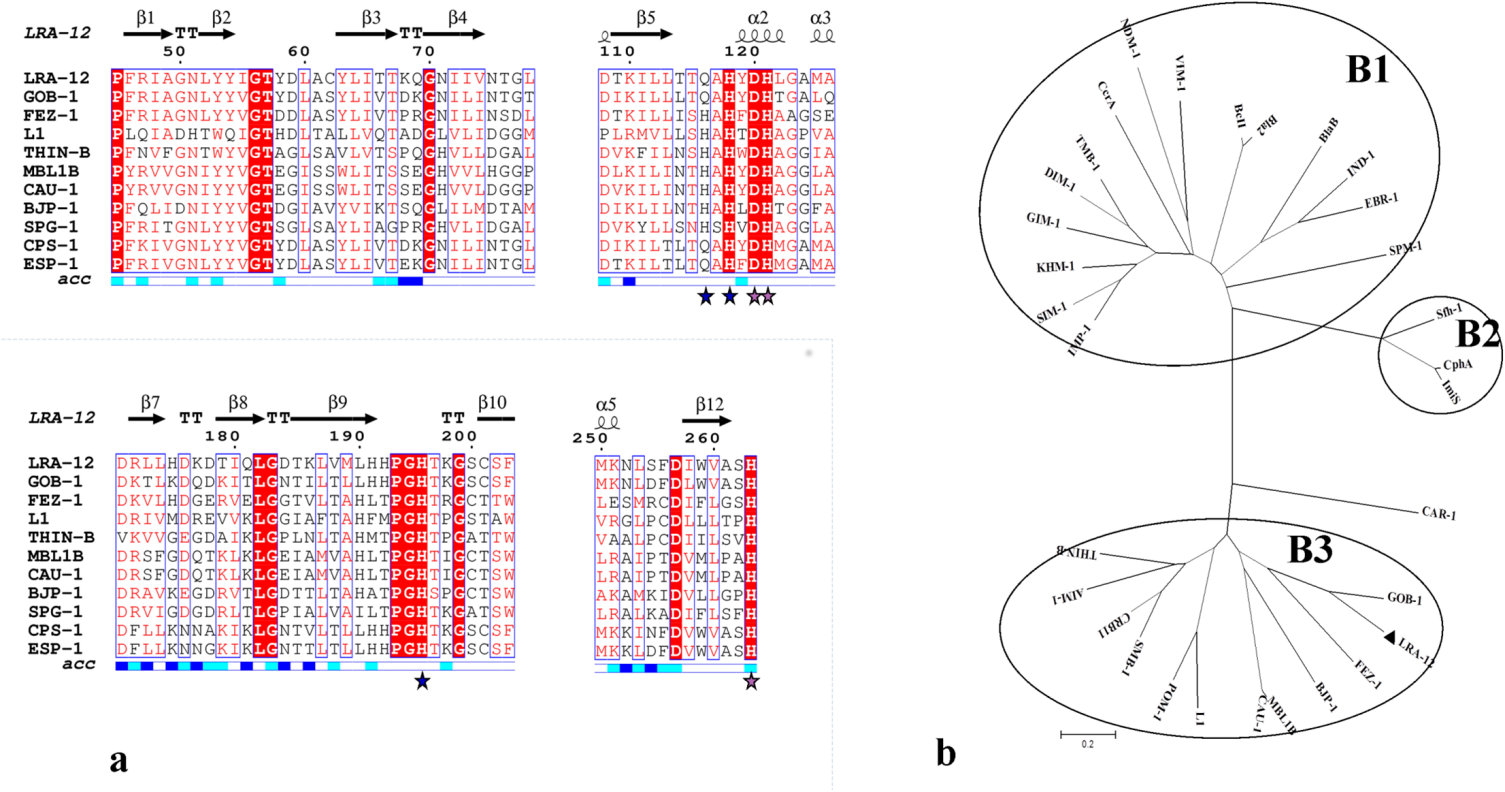
Sequence analysis on LRA-12 protein. (a) Multi-alignment of amino acid sequences of LRA-12 and other representative class B3 β-lactamases, using the class B standard numbering scheme. Only the four more conserved segments of the sequences are shown for easier visualization. Location of α-helices and β-sheets is indicated in the upper side (taken from the PDB file), and relative solvent accessibility in the bottom (blue: highly accessible; cyan: poorly accessible; white: hidden or non-accessible). Blue and pink stars indicate the position of conserved residues in metal-binding sites 1 and 2, respectively (see text for further details). The figure was prepared using Espript (http://espript.ibcp.fr/ESPript/ESPript/). (b) Neighbor joining tree constructed using class B β-lactamases sequences from the three different sub-classes.

The predicted amino acid sequence of the metagenomic metallo-β-lactamase (MBL) LRA-12 from Alaskan soil revealed a high percentage amino acid identity with previously described MBLs from environmental origin. It presented 60–69% amino acid identity with GOB MBLs from *Elizabethkingia meningoseptica* (formerly known as *Chryseobacterium meningosepticum*) [47], including GOB-18 whose structure has been recently solved [48], with which LRA-12 seems to be evolutionarily close (Fig 1).

The MBL also showed 41% and 33% identity with FEZ-1 (*Fluoribacter gormanii*) and BJP-1 (*Bradyrhizobium japonicum*), respectively, two class B3 enzymes for which the crystallographic structures have been solved [49, 50].

### Resistance phenotype conferred by LRA-12 in *E*. *coli* is comparable to clinical pathogens with acquired metallo-β-lactamases

Compared to the reference *Escherichia coli* EPI300 strain (Table 1), the clones producing the LRA-12 β-lactamase cloned in the fosmid pCC1FOS exhibit a susceptibility behavior similar to that conferred by acquired metallo-β-lactamases commonly associated with clinical pathogens, such as VIM, IMP, and to other non-class B1 enzymes when produced in *E*. *coli* clones [10]. The *E*. *coli* EPI300 clone expressing LRA-12 was resistant to amoxicillin, amoxicillin/clavulanate, cephalothin, cefoxitin, and oxyimino-cephalosporins (cefotaxime, ceftazidime and cefepime). For carbapenems, the clone was also resistant to meropenem (MIC value in the break point for resistance: 4 μg/mL), but susceptible to imipenem, although the MIC value for this drug (1 μg/mL) was about 16/32-fold higher than the MIC for recipient *E*. *coli* control strains (Table 1) and, as observed in *E*. *coli* strains producing other MBLs, resistance to carbapenems could be observed only when a high inoculum is present [51]. As expected for a class B β-lactamase, the clone showed low resistance to the monobactam aztreonam. In addition, *E*. *coli* EPI300/pCC1FOS-*bla*_LRA-12_ showed positive synergy between EDTA and carbapenem-containing disks in a disk diffusion test (data not shown).

**Table 1:** Minimum inhibitory concentrations (in μg/mL) of recombinant *E*. *coli* producing LRA-12 β-lactamase.

Antibiotics tested	*E*. *coli* EPI300	*E*. *coli* EPI300 / pCC1FOS	*E*. *coli* EPI300 / pCC1FOS-*bla*_LRA-12_ ^a^
Amoxicillin	4	4	256
Amoxicillin/clavulanic acid	4/2	4/2	128/64
Cephalothin	4	8	256
Ceftazidime	0.125	0.25	32
Cefotaxime	0.032	0.032	32
Cefepime	0.016	0.016	16
Cefoxitin	2	2	32
Imipenem	0.063	0.032	1
Meropenem	0.032	0.032	4
Aztreonam	0.125	0.063	1
Cloramphenicol	0.25	32	32

^a^ This clone is equivalent to the βLR12 clone in reference [33].

### LRA-12 displays a strong carbapenemase activity

The kinetic parameters of LRA-12 were determined for a representative group of β-lactams (Table 2). Results show that LRA-12 exhibits a broad spectrum profile, since penicillins, cephalosporins, cephamycins and even carbapenems were hydrolyzed, with *k*_cat_/*K*_m_ values ranging from 0.16 to 1.75 μM^-1^.s^-1^. The enzyme exhibited higher apparent affinity for cephalothin, cefuroxime and cefoxitin (lowest *K*_m_ values; 11, 20 and 4 μM, respectively). For cephalothin and cefoxitin, accompanying low turnover rates (18 and 7 s^-1^, respectively) result in the highest catalytic efficiency values, equivalent to that of LRA-12 acting upon imipenem. High *k*_cat_/*K*_m_ values were also observed for LRA-12 acting upon all three carbapenems evaluated, imipenem, meropenem and ertapenem (1.69, 0.84 and 1.33 μM^-1^.s^-1^, respectively), in contrast to its behavior with oxyimino-cephalosporins, with which it displayed lower catalytic efficiencies (0.20, 0.19 and 0.16 μM^-1^.s^-1^ for ceftazidime, cefotaxime and cefepime, respectively). As expected for metallo-β-lactamases [12], aztreonam inhibited LRA-12, with a *K*_i_ of 146 μM.

**Table 2:** Kinetic parameters of LRA-12 metallo-β-lactamase.

β-Lactam	*K*_m_ (μM)	*k*_cat_ (s^-1^)	*k*_cat_/*K*_m_ (μM^-1^.s^-1^)	Relative *k*_cat_/*K*_m_ (%)^a^
**Imipenem**	39 ± 7	66 ± 5	1.69 ± 0.42	100
**Meropenem**	113 ± 20	95 ± 11	0.84 ± 0.23	49.7
**Ertapenem**	43 ± 6	57 ± 4	1.33 ± 0.27	78.7
**Benzyl-penicillin**	47 ± 4	70 ± 2	1.49 ± 0.15	88.2
**Ampicillin**	183 ± 45	75 ± 11	0.41 ± 0.14	24.3
**Piperacillin**	83 ± 9	74 ± 3	0.89 ± 0.13	52.7
**Cephalothin**	11 ± 2	18 ± 0.8	1.64 ± 0.37	97
**Cefuroxime**	20 ± 3.4	16 ± 0.9	0.80 ± 0.19	47.3
**Ceftazidime**	59 ± 8	12 ± 0.8	0.20 ± 0.04	11.8
**Cefotaxime**	36 ± 4	7 ± 0.3	0.19 ± 0.03	11.2
**Cefepime**	93 ± 13	15 ± 1.4	0.16 ± 0.04	9.5
**Cefoxitin**	4 ± 0.6	7 ± 0.4	1.75 ± 0.32	103.5

^a^ Catalytic efficiency of LRA-12 on various β-lactams compared with imipenem.

Kinetic parameters were compared to those reported for other class B3 metallo-β-lactamases (Table 3). Metagenomic LRA-12 β-lactamase presents a carbapenemase behavior similar to the closely related GOB β-lactamases, especially GOB-18 [47, 52]. In this sense, both LRA-12 and GOB enzymes present the highest carbapenemase activity among class B3 MBLs. Also, the activity of LRA-12 with penicillins was also similar to GOB β-lactamases, whereas the displayed activity against cephaloporins was less conserved. Cephalothin and cefoxitin were apparently better substrates for LRA-12 than for other class B3 enzymes (with the exception of FEZ-1 against cephalothin). Oxyimino-cephalosporins, cefotaxime and ceftazidime, behaved in contrasting manners: LRA-12 hydrolyzed ceftazidime at up to a 95-fold higher rate than did FEZ-1, BJP-1 and CAU-1, although LRA-12 was 4-fold less efficient than GOB-1; in contrast, cefotaxime was a poor substrate for both LRA-12 and BJP-1 [50].

**Table 3:** Comparative catalytic efficiencies of LRA-12 and other class B3 metallo-β-lactamases.

	*k*_cat_/*K*_m_ (μM^-1^.s^-1^) for MBL:					
Substrate	LRA-12	GOB-1 [47]	GOB-18 [52]	FEZ-1 [53]	BJP-1 [50]	CAU-1 [54]
**Benzylpenicillin**	1.49	1.87	2.1	0.11	0.13	0.45
**Ampicillin**	0.41	0.35	ND	0.011	0.019	0.50
**Cephalothin**	1.64	0.67	0.95^a^	2.5	0.58	0.43
**Cefoxitin**	1.75	0.25	ND	0.27	0.071	ND
**Ceftazidime**	0.19	0.76	ND	0.004	0.0043	0.002
**Cefotaxime**	0.2	0.85	0.94	2.4	0.14	ND
**Imipenem**	1.69	0.66	1.6	0.2	0.06	0.2
**Meropenem**	0.84	5.34	1.8	0.5	0.83	0.26

^a^ Cephaloridine was tested instead of cephalothin. ND: not determined.

### Chelating agents inactivate LRA-12 with different efficiencies

Activity of LRA-12 in presence of two different chelating agents was assessed by two approaches.

**Fig 2:**
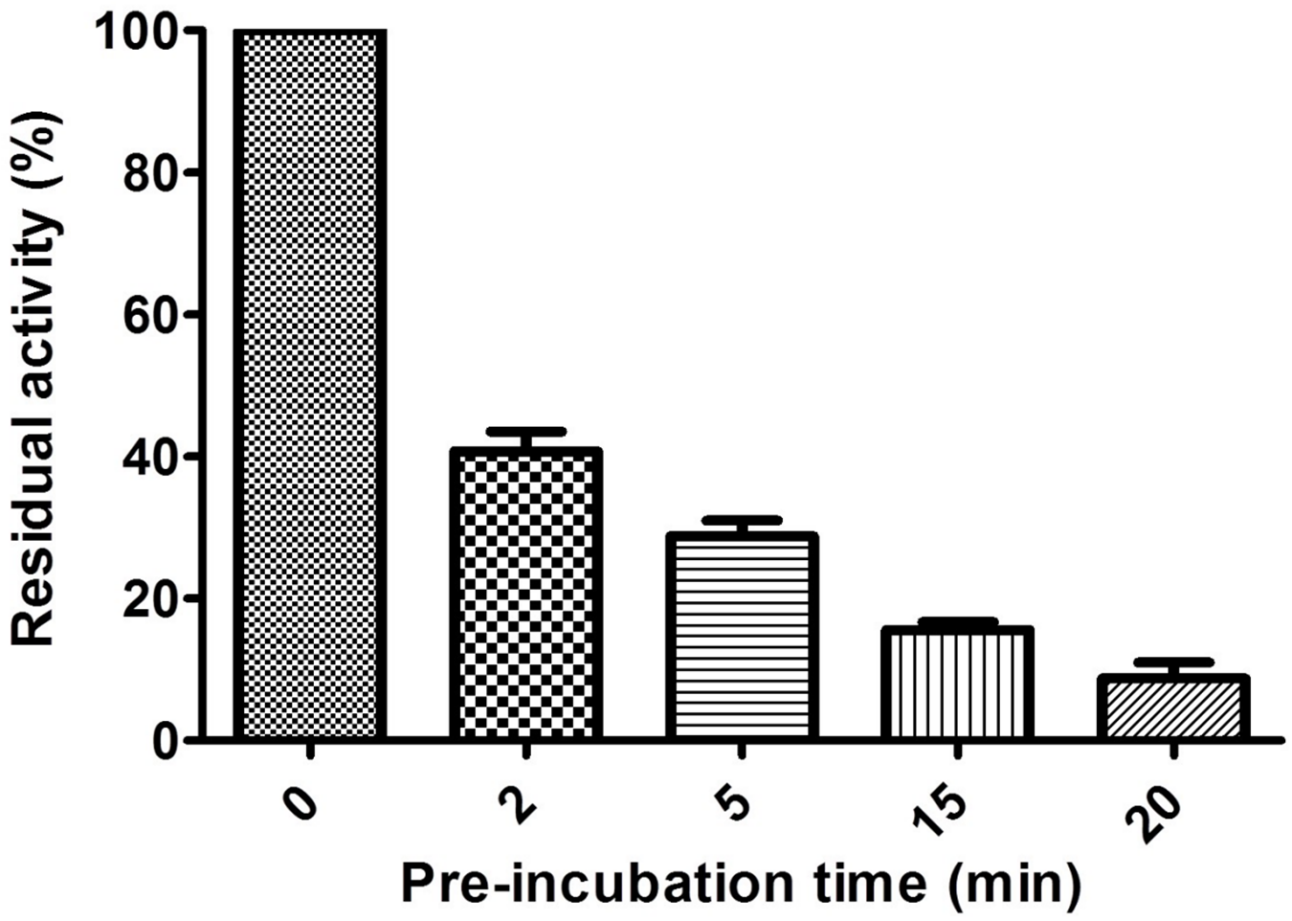
Influence of pre-incubation with EDTA on the residual activity of LRA-12.

Pre-incubation of LRA-12 with 50 mM EDTA inactivate 91% of the enzyme within 20 min (Fig 2) and EDTA did not inhibit enzyme activity when added simultaneously. For DPA (used at 1 mM), the enzyme’s activity was completely lost even when mixed simultaneously with the reporter (data not shown).

EDTA displayed an IC_50_ of 113 μM. The IC_50_ for DPA was 460 μM. These results suggest that DPA was a stronger inhibitor than EDTA, as DPA did not require pre-incubation; however, the IC_50_ value for EDTA was lower than that for DPA.

### Overall structure of LRA-12 metallo-β-lactamase

Metagenomic LRA-12 crystallized in space group P 1 2_1_ 1, and crystals diffracted at a final resolution of 2.1 Å. Main data and refinement statistics are shown in Table 4. It is important to note that, at the time this structure was solved and deposited (2015/08/27; released 2015/09/16), the closest structures available at the PDB were FEZ-1 (PDB 1JT1) and BJP-1 (2GMN) β-lactamases, with 41% and 33% amino acid identity, respectively (see above), for which FEZ-1 coordinates were used for molecular replacement analysis and phasing process; recently, the structure of GOB-18 was also released (PDB 5K0W; released 2016/08/03), and this enzyme shares 61% amino acid identity with LRA-12, making it the closest structural match [48].

**Table 4:** X-Ray data collection and refinement statistics for LRA-12 β-lactamase.

Crystal	LRA-12
**PDB code**	5aeb
**Data Collection:**	
***Space group***	P 1 2_1_ 1
***Cell parameters (Å)***	a = 47.15; b = 80.62; c = 78.29; α = 90.00; β = 98.83; γ = 90.00
***Average mosaicity***	0.17
***Subunits/asymmetric unit***	2
***Resolution range (Å)*^a^**	46.59–2.10 (2.21–2.10)
***No*. *of unique reflections***	33,531
***R***_***merge***_ ***(%)*^a^^,^^b^**	11.5 (48.6)
***Redundancy*^a^**	3.5 (3.5)
***Completeness (%)*^a^**	99.0 (98.2)
***<I>/<σI>*^a^**	7.2 (3.3)
**Refinement:**	
***Resolution range***	42.9–2.10
***Number of protein atoms***	4,160
***Number of water molecules***	232
***R***_***cryst***_ ***(%)***	18.4
***R***_***free***_ ***(%)***	23.5
***RMS***^***b***^ ***deviations from ideal stereochemistry*:**	
*bond lengths (Å)*	0.012
*bond angles (*^*o*^*)*	1.471
***Mean B factor (all atoms) (Å***^***2***^***)***	26.5
***Ramachandran plot*:**	
*Favored region (%)*	96.4
*Allowed regions (%)*	3.4
*Outlier regions (%)*	0.2

^a^ Statistics for the highest resolution shell are given in parentheses.

^b^ RMS: Root-mean square; MSD: Root mean square difference.

The refined structure consists in two monomers per asymmetric unit. Monomer A includes 264 amino acids of the 269 residue long mature β-lactamase, from Val28 to Asn306; monomer B is composed of 266 residues, from Gln26 to Asn306, following BBL numbering [11]. Both monomers have 30% helical content (14 helices; 79 residues with 61 residues in α-helices and 18 in 3–10 helices) and 23% β-sheet composition (16 strands; 60 residues). The structure is solvated by 232 ordered water molecules.

**Fig 3:**
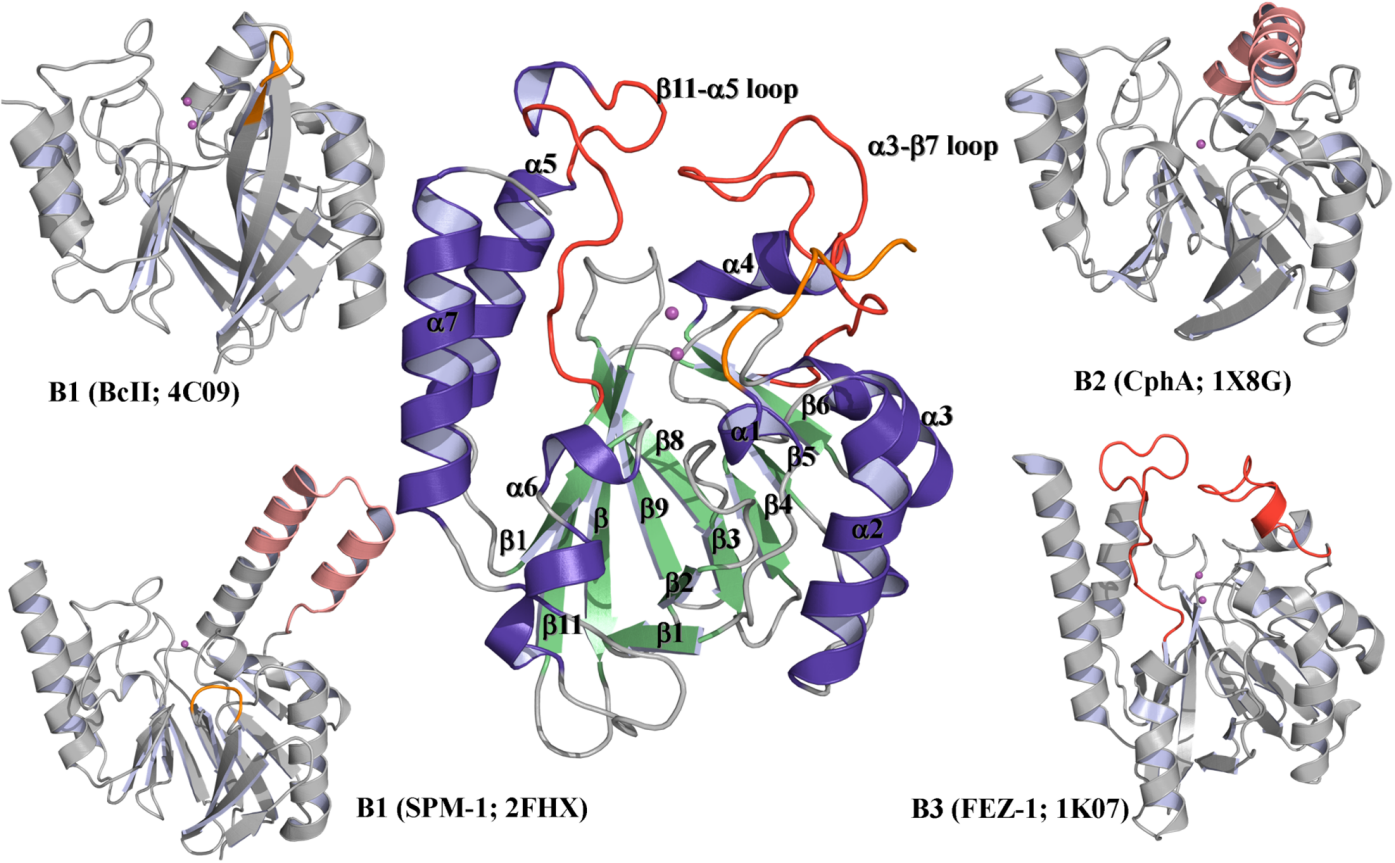
Comparative analysis of overall structures of LRA-12 (central structure) and other MBLs. Color codes: purple: α-helices; green: β-sheets (for LRA-12); pink spheres: Zn(II) atoms; red loops: elongated α3-β7 and β11-α5 loops in B3 β-lactamases; orange: N-terminal segment of LRA-12, and short mobile loops in BcII and SPM-1; pink α-helices: elongated α-helix and extended α3-α4 helix in CphA and SPM-1, respectively. For further details, see reference [10].

The overall structure of LRA-12 conserves the main structural features of a class B β-lactamase [55], showing an αβ/βα sandwich in which α-helices are exposed to the solvent and surround a compact core of β-sheets (Fig 3).

The root mean-square deviation (rmsd) between the equivalent Cα atoms in both monomers is 0.31 Å and no significant difference is found between the two active sites. Due to this observation, further discussion will refer to both monomers unless otherwise noted.

LRA-12 structure is very similar to other class B3 β-lactamases, despite low sequence identities within the sub-class [48, 49, 56]. The overall fold is similar to those found in other B3 enzymes from environmental origin like FEZ-1, BJP-1, GOB-18 and L1 (Fig 3). Superimposition of the common Cα atoms with other class B3 MBLs resulted in RMSDs of 1.35 Å (97% coverage; cov.), 1.49 Å (96% cov.), 1.42 Å (98% cov.), and 1.63 Å (98% cov.), compared to FEZ-1 (PDB 1JT1), BJP-1 (PDB 2GMN), L1 (PDB 2FM6), and SMB-1 (PDB 3VPE), respectively. The highest structural similarity was obtained with GOB-18 (PDB 5K0W), with RMSD of 0.76 Å.

A 23-residue-long loop (Asp148-Arg172) with apparent high mobility, between α3 and β7, is present at the entrance of the active site. This loop is also found in other B3 β-lactamases, although some of them like GOB-18, FEZ-1 and BJP-1 include a short α-helix within this loop (Fig 3). There is another long loop connecting β11 and α5 (Asn220-Pro237) also present in other enzymes like FEZ-1, BJP-1 and L1, but in LRA-12 the loop is interrupted by a short α-helix (Lys229-Val233). These two loops could be relevant for the shape and dimensions of the active site. The α3-β7 loop could also influence the access to the active site cavity, because of its location as a “flexible lid” partially covering the active site.

An important difference between LRA-12 and other B3 enzymes like FEZ-1, L1 and BJP-1 is the absence of disulfide bridges in LRA-12. In FEZ-1 and L1, a disulfide bridge occurs between Cys256 and Cys290 (FEZ-1) or Cys296 (L1); these cysteines are replaced by aromatic residues Phe256 and Tyr290, respectively. Also, Cys181 and Cys201, creating the disulfide bridge in BJP-1, are replaced by Ser200 and Asn220, respectively, in LRA-12. Metagenomic β-lactamase LRA-12 has only two cysteine residues not involved in disulfide bridges: Cys70 (Ser70 and Ser54 in FEZ-1 and GOB-18, respectively); and Cys201, also present in GOB-18 (Cys180), which is buried within the β-lactamase core and associated through hydrogen bonds with backbone carbonyl groups of Thr114 and Thr115, located at the β5-α3 loop.

Interestingly, a cobalt ion was modeled between the two monomers of LRA-12 in the asymmetric unit, bound to Glu140-Oε and His175-Nε2 in a tetrahedral coordination. This cobalt, present in the crystallization buffers, could help in the crystallization process by stabilizing intermolecular interactions, as previously suggested [57].

Finally, five sulfate ions (2 in monomer A and 3 in monomer B) were also modeled, all bound to solvent-exposed residues and not interfering with important residues from the active site cavity.

### Metal coordination and active site of LRA-12

The active site of LRA-12, as in the other metallo-β-lactamases, is located between the two “αβ” moieties of the αββα fold, above the β-sheets, covered by the flexible α3-β7 loop, and contoured by the 10 amino acids N-terminal segment, the β11-α5 loop, and the α4. The catalytic cavity has a tridimensional geometry of a shallow cleft having 150–170 Å^3^, similar to what was reported for GOB-18 (150–200 Å^3^) [48]. The volume of the LRA-12 cavity seems to be higher than other B3 β-lactamases like FEZ-1 (ca. 120 Å^3^), BJP-1 (ca. 110 Å^3^), and L1 (ca. 70 Å^3^). These findings are in agreement with the aforementioned discussion about the equivalent catalytic behavior of LRA-12 and GOB β-lactamases, compared to other B3 β-lactamases [5, 10, 52], and could be related to the observed kinetic behavior, especially the high carbapenemase activity compared to other class B3 metallo-β-lactamases.

The β-lactamase LRA-12 contains two heavy atoms in the active site of each of the two monomers in the asymmetric unit, according to well-defined electron densities. This is consistent with the fact that most of the class B3 β-lactamases contain two zinc ions in the active site, constituting what is known as the metal- or zinc-binding site. Comparative constitution of zinc-binding sites with other MBLs is shown in Table 5.

**Table 5:** Composition of the metal-binding sites in LRA-12 and other metallo-β-lactamases.

MBL	Metal site 1	Metal site 2	221^b^	#Zn^c^
**B3 LRA-12**	Gln116-His118-His196	Asp120-His121-His263	Met	2
**B3 GOB-18**^a^	Gln98-His100-His175	Asp102-His103-His241	Met	2
**B3 (FEZ-1)**	His116-His118-His196	Asp120-His121-His263	Ser	2
**B1**	His116-His118-His196	Asp120-Cys221-His263	Cys	2
**B2**	Asp120-Cys221-His263	His118-Met146 or His196(?)	Cys	1/2

^a^ PDB numbering, equivalent to positions 116, 118, 196; 120, 121, 263, in other B3 enzymes.

^b^ Met200 in GOB-18 (PDB 5K0W).

^c^ Number of Zn(II) atoms.

**Fig 4:**
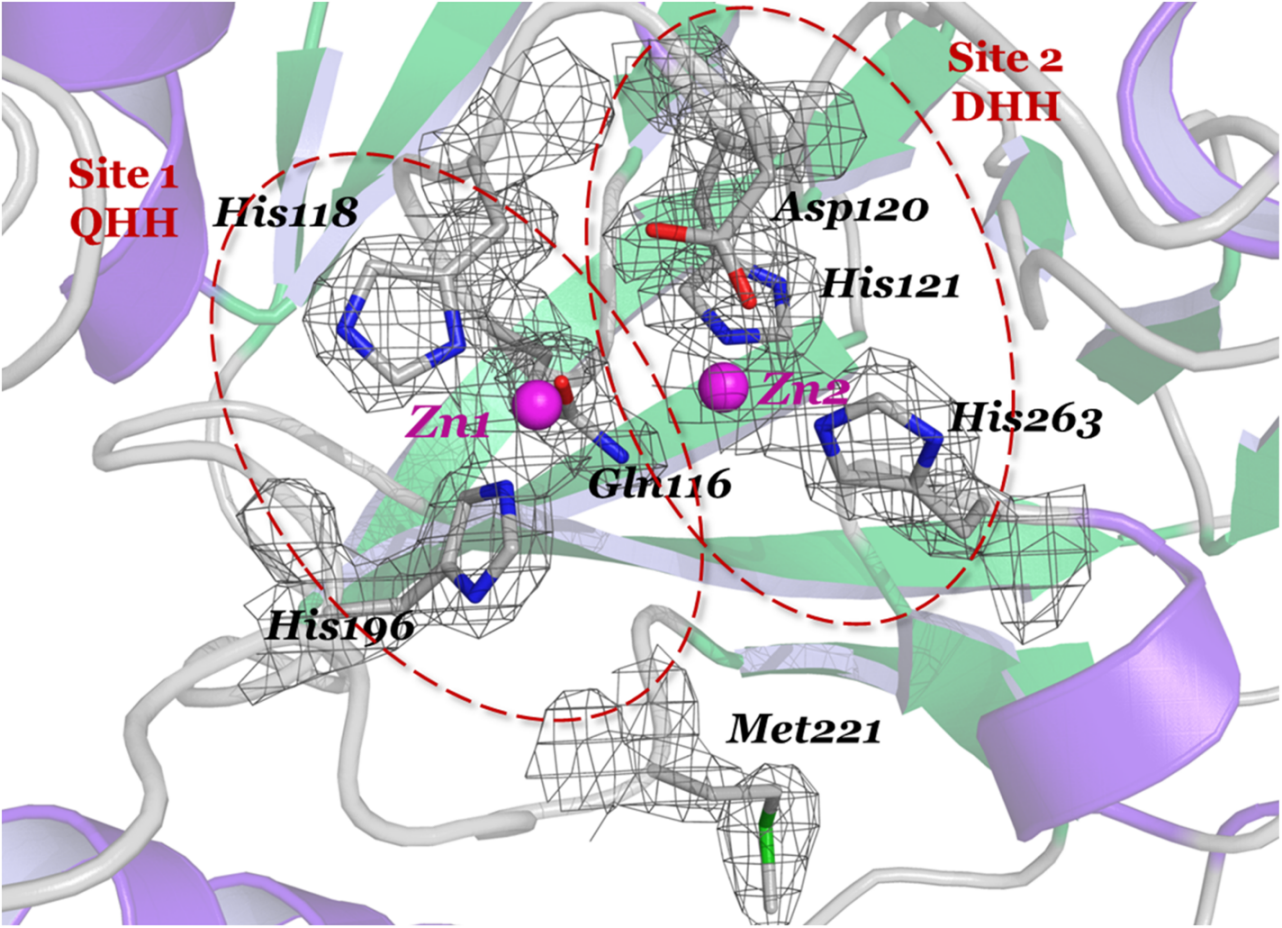
Detail of the active site of LRA-12 β-lactamase. The *2F*_*0*_ *–F*_*c*_ map was contoured at 1.5 σ (in grey) around the most important amino acid residues that are part of the metal-binding sites in the active site cavity: Gln116-His118-His196 (Site 1; QHH), and Asp120-His121-His263 (Site 2; DHH). Zinc ions (Zn1 and Zn2) are shown as magenta spheres.

**Fig 5:**
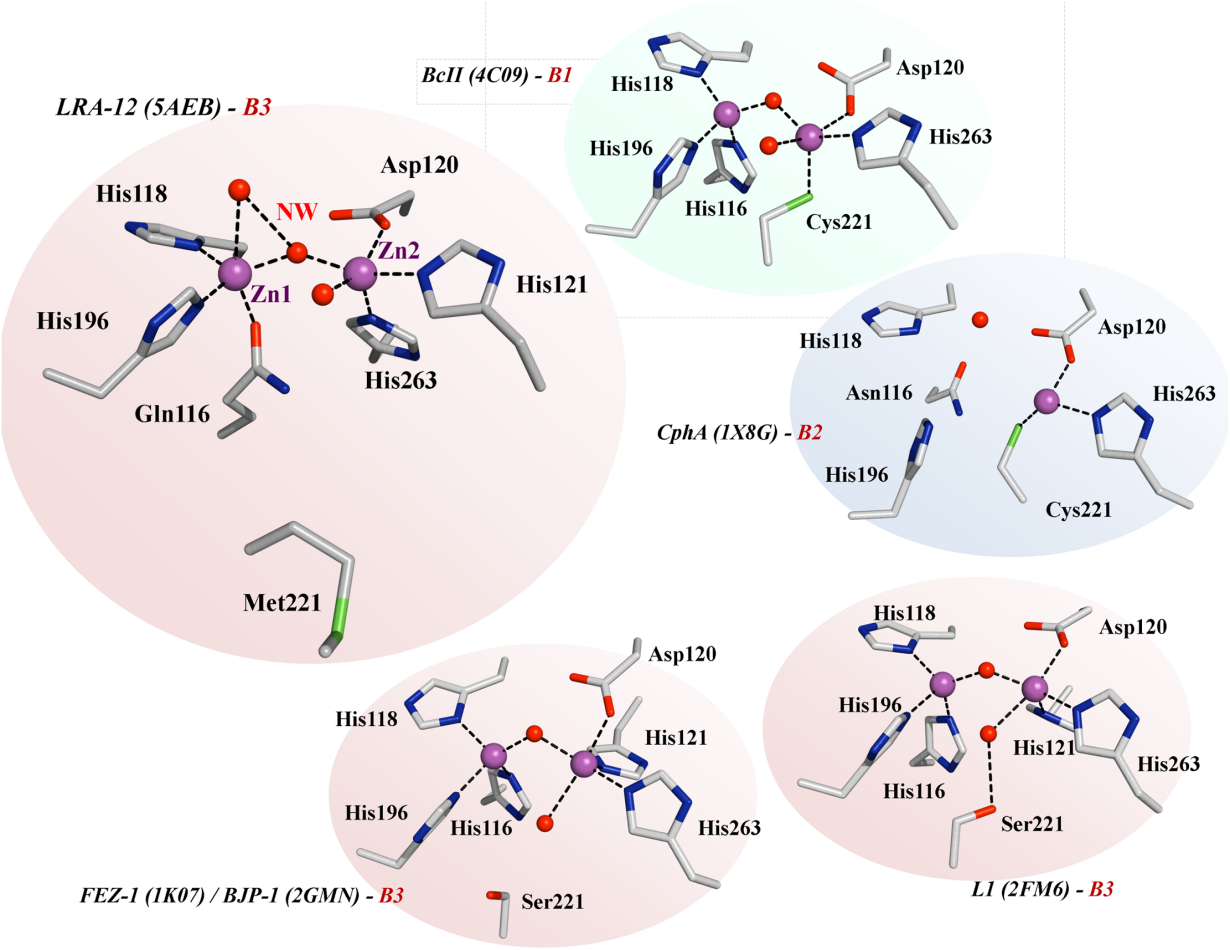
Comparative view of the metal-binding sites of LRA-12 and other metallo-β-lactamases. Zinc ions (Zn1 and Zn2) and water molecules are represented as magenta and red spheres, respectively; NW, nucleophilic water. Black dotted lines represent the hydrogen bonding interactions between residues or atoms. For ease of interpretation, Zn(II) and water molecules have the same spatial orientation in all figures.

The metal-binding site 1 is defined by Gln116, His118, and His196 (site QHH), which is uniquely found in this enzyme and in GOB β-lactamases [48]; zinc-binding site 2 is constituted by Asp120, His121, and His263 (site DHH) (Fig 4). Both Zn(II) ions adopt a distorted squared pyramidal molecular geometry, in which three water molecules, along with the aforementioned conserved residues, participate in the metal-coordination. This geometry is similar in GOB-18 [48], and different to what is observed in the other B3 β-lactamases crystallized, in which the coordination sphere in the zinc-binding site 2 adopts a trigonal bipyramidal geometry with two water ligands [49, 56, 58] (Fig 5).

The zinc ion at the QHH site (Zn1308 in the PDB) is bound to Gln116-Oε1, His118-Nδ1, His196-Nε2, and two water molecules, Wat2045 and Wat2040 in the PDB coordinate file. The latter constitutes the nucleophilic water molecule that serves as a bridge between both Zn(II) ions, as observed in most of the class B3 MBLs, providing one of the binding points with zinc ion at DHH site (Zn1307 in the PDB), which is also bound to Asp120-Oδ2, His121-Nε2, His263-Nε2, and water molecule Wat2041 (Fig 5).

Also, intra- and inter-metal site interactions between residues from both metal sites also exist: Asp120-Oδ2 with His263-Nε2 (site 2 –site 2), His118-O with Asp120-N (site 1 –site 2), His118-O with His121-N (site 1 –site 2), and His121-Nε2 with His263-Nε2 (site 2 –site 2).

Both Zn(II) ions are separated by a Zn-Zn distance of 3.60 and 3.66 Å in monomer A and B, respectively, which is equivalent to distances observed in other dinuclear B3 MBLs [48, 49, 56, 58].

Active site residues participating in the direct coordination of Zn(II) ligands are also stabilized by hydrogen bonds with outer sphere residues. In zinc-binding site 1, Gln116-Nε2 interacts with Asn220-Nδ2 and a water molecule, His118-Nε2 with a water molecule, and His196-Nδ1 with Thr223-Oγ1. In metal-binding site 2, His263-Nδ1 interacts with Asp67-Oδ2, and His263-O with Ser265-N and Gln266-N (not shown).

A remarkable difference between LRA-12 and other class B β-lactamases is the presence of a methionine residue at position 221 that is not involved in the coordination of the metal-binding site. As shown in Table 5 and Fig 5, Met221 replaces Cys221 in known structures of sub-class B1 and B2 enzymes, and Ser221 in other B3 metallo-β-lactamases (except GOB-18 which also displays a methionine in position 221). This substitution results in different outcomes on the protein’s structure. Cysteine 221 is directly bound to a zinc atom in class B1 and B2 β-lactamases. As shown in *S*. *maltophilia*’s L1 (and probably FEZ-1 and BJP-1), Ser221 does not directly interact with the Zn(II) but with a second water molecule in the active site which could act as proton donor during catalysis [59]; instead of this, Ser221 is turned opposite in FEZ-1 and BJP-1 (Fig 5).

In LRA-12, a water molecule (Wat2042) occupies the position of Ser221 lateral chain in FEZ-1/BJP-1, forcing the side chain of Met221 to point outwards from the active site, which enables a displacement of the β11-α5 loop (containing Met221) that carries it, thus increasing the size of the cavity; compared to FEZ-1, BJP-1, and L1, the Cα of residue at 221 is displaced 2.70, 2.72 and 3.06 Å, respectively.

As in GOB-18, Met221 of LRA-12 is located within a predominantly hydrophobic cavity, without any direct interaction with the catalytic residues, and its role has been postulated to be important in protein stability [60]. Moreover, the differential position of Met221 in LRA-12 could contribute to an overall change in the geometry of the active site cavity.

## Conclusions

In this study, we report the phenotypic, kinetic and structural analysis of a metallo-β-lactamase isolated by metagenomics from an uncultured bacterium from Alaskan soils.

LRA-12 is a class B3, di-zinc dependent metallo-β-lactamase with highest amino acid identity to the *Elizabethkingia meningoseptica* GOB enzymes. This subclass includes mostly chromosomally-encoded β-lactamases from environmental microorganisms, although they have also been isolated occasionally in clinical settings (e.g. *Stenotrophomonas maltophilia* producing L1).

Our results support the hypothesis that many β-lactamases originate on bacterial chromosomes. There is an increasing frequency of reports that provide evidence that the resistome contained in environmental microorganisms is a source of enzymes with native wide-range activity spectrum towards most β-lactams, even before being recruited and disseminated among human pathogens.

Metallo-β-lactamases are part of a vast family of proteins with a stable scaffold that were able to evolve divergently toward diverse activities, and this evolution relied on the variation in the sequence and length of specific loops and other domains that enabled adaptation of their catalytic cavities to different types of substrates, expanding their functions within the Bacteria as well as the Eukaryotes.

The environmental metagenome is a rich source of MBLs presenting both biochemical and structural similarities to other widely distributed clinically-relevant carbapenemases.

The structure of LRA-12 suggests that MBLs occur in environments independent of local antibiotic use. The high use of antibiotics in clinical settings as well as in livestock activities likely favored the recruitment of “silent” genes contained in culturable or unculturable microorganisms in a specific environment, and are prone of being disseminated to pathogens by horizontal gene transfer. The *bla*_LRA-12_ gene is embedded in a ~30 kb genetic platform lacking any putative recombination/transposition signal (data not shown), which, considering that the metagenomic sample comes from an area with low exposure to antibiotics introduced by people, the possibility of occurrence of gene exchanges and selection could still be low.

## References

[1] Khan AU, Nordmann P. Spread of carbapenemase NDM-1 producers: the situation in India and what may be proposed. Scand J Infect Dis. 2012;44(7):531–5. doi: 10.3109/00365548.2012.669046 22497308 10.3109/00365548.2012.669046

[2] Nordmann P, Poirel L, Walsh TR, Livermore DM. The emerging NDM carbapenemases. Trends Microbiol. 2011;19(12):588–95. doi: 10.1016/j.tim.2011.09.005 22078325 10.1016/j.tim.2011.09.005

[3] da Silva RM, Traebert J, Galato D. Klebsiella pneumoniae carbapenemase (KPC)-producing *Klebsiella pneumoniae*: a review of epidemiological and clinical aspects. Expert opinion on biological therapy. 2012;12(6):663–71. doi: 10.1517/14712598.2012.681369 22506862 10.1517/14712598.2012.681369

[4] Canton R, Coque TM. The CTX-M β-lactamase pandemic. Curr Opin Microbiol. 2006;9(5):466–75. doi: 10.1016/j.mib.2006.08.011 16942899 10.1016/j.mib.2006.08.011

[5] Gutkind GO, Di Conza J, Power P, Radice M. β-Lactamase-mediated resistance: a biochemical, epidemiological and genetic overview. Curr Pharm Des. 2013;19(2):164–208. 22894615

[6] Poirel L, Naas T, Nordmann P. Diversity, epidemiology, and genetics of class D β-lactamases. Antimicrob Agents Chemother. 2010;54(1):24–38. doi: 10.1128/AAC.01512-08 19721065 10.1128/AAC.01512-08 PMC2798486

[7] Opazo A, Dominguez M, Bello H, Amyes SG, Gonzalez-Rocha G. OXA-type carbapenemases in *Acinetobacter baumannii* in South America. J Infect Dev Ctries. 2012;6(4):311–6. 22505439 10.3855/jidc.2310

[8] Nordmann P, Naas T, Poirel L. Global spread of Carbapenemase-producing *Enterobacteriaceae*. Emerg Infect Dis. 2011;17(10):1791–8. doi: 10.3201/eid1710.110655 22000347 10.3201/eid1710.110655 PMC3310682

[9] Daiyasu H, Osaka K, Ishino Y, Toh H. Expansion of the zinc metallo-hydrolase family of the β-lactamase fold. FEBS Lett. 2001;503(1):1–6. 11513844 10.1016/s0014-5793(01)02686-2

[10] Bebrone C. Metallo-β-lactamases (classification, activity, genetic organization, structure, zinc coordination) and their superfamily. Biochem Pharmacol. 2007;74(12):1686–701. doi: 10.1016/j.bcp.2007.05.021 17597585 10.1016/j.bcp.2007.05.021

[11] Galleni M, Lamotte-Brasseur J, Rossolini GM, Spencer J, Dideberg O, Frere JM. Standard numbering scheme for class B β-lactamases. Antimicrob Agents Chemother. 2001;45(3):660–3. doi: 10.1128/AAC.45.3.660-663.2001 11181339 10.1128/AAC.45.3.660-663.2001 PMC90352

[12] Palzkill T. Metallo-β-lactamase structure and function. Ann N Y Acad Sci. 2013;1277:91–104. doi: 10.1111/j.1749-6632.2012.06796.x 23163348 10.1111/j.1749-6632.2012.06796.x PMC3970115

[13] Bonnet R. Growing group of extended-spectrum β-lactamases: the CTX-M enzymes. Antimicrob Agents Chemother. 2004;48(1):1–14. doi: 10.1128/AAC.48.1.1-14.2004 14693512 10.1128/AAC.48.1.1-14.2004 PMC310187

[14] Humeniuk C, Arlet G, Gautier V, Grimont P, Labia R, Philippon A. β-Lactamases of *Kluyvera ascorbata*, probable progenitors of some plasmid-encoded CTX-M types. Antimicrob Agents Chemother. 2002;46(9):3045–9. doi: 10.1128/AAC.46.9.3045-3049.2002 12183268 10.1128/AAC.46.9.3045-3049.2002 PMC127423

[15] Olson AB, Silverman M, Boyd DA, McGeer A, Willey BM, Pong-Porter V, et al Identification of a progenitor of the CTX-M-9 group of extended-spectrum β-lactamases from *Kluyvera georgiana* isolated in Guyana. Antimicrob Agents Chemother. 2005;49(5):2112–5. doi: 10.1128/AAC.49.5.2112-2115.2005 15855541 10.1128/AAC.49.5.2112-2115.2005 PMC1087624

[16] Poirel L, Kampfer P, Nordmann P. Chromosome-encoded Ambler class A β-lactamase of *Kluyvera georgiana*, a probable progenitor of a subgroup of CTX-M extended-spectrum β-lactamases. Antimicrob Agents Chemother. 2002;46(12):4038–40. doi: 10.1128/AAC.46.12.4038-4040.2002 12435721 10.1128/AAC.46.12.4038-4040.2002 PMC132763

[17] Rodriguez MM, Power P, Radice M, Vay C, Famiglietti A, Galleni M, et al Chromosome-encoded CTX-M-3 from *Kluyvera ascorbata*: a possible origin of plasmid-borne CTX-M-1-derived cefotaximases. Antimicrob Agents Chemother. 2004;48(12):4895–7. doi: 10.1128/AAC.48.12.4895-4897.2004 15561876 10.1128/AAC.48.12.4895-4897.2004 PMC529199

[18] Rodriguez MM, Power P, Sader H, Galleni M, Gutkind G. Novel chromosome-encoded CTX-M-78 β-lactamase from a *Kluyvera georgiana* clinical isolate as a putative origin of CTX-M-25 subgroup. Antimicrob Agents Chemother. 2010;54:3070–1. doi: 10.1128/AAC.01615-09 20421403 10.1128/AAC.01615-09 PMC2897315

[19] Gudeta DD, Bortolaia V, Amos G, Wellington EM, Brandt KK, Poirel L, et al The soil microbiota harbors a diversity of carbapenem-hydrolyzing β-lactamases of potential clinical relevance. Antimicrob Agents Chemother. 2015;60(1):151–60. doi: 10.1128/AAC.01424-15 26482314 10.1128/AAC.01424-15 PMC4704184

[20] D'Costa VM, McGrann KM, Hughes DW, Wright GD. Sampling the antibiotic resistome. Science (New York, NY. 2006;311(5759):374–7. doi: 10.1126/science.1120800 16424339 10.1126/science.1120800

[21] Wright GD. The antibiotic resistome. Expert Opin Drug Discov. 2010;5(8):779–88. doi: 10.1517/17460441.2010.497535 22827799 10.1517/17460441.2010.497535

[22] Galan JC, Gonzalez-Candelas F, Rolain JM, Canton R. Antibiotics as selectors and accelerators of diversity in the mechanisms of resistance: from the resistome to genetic plasticity in the β-lactamases world. Front Microbiol. 2013;4:9 doi: 10.3389/fmicb.2013.00009 23404545 10.3389/fmicb.2013.00009 PMC3567504

[23] Handelsman J, Liles M, Mann D, Riesenfeld C, Goodman RM. Cloning the metagenome: culture-independent access to the diversity and functions of the uncultivated microbial world Methods in Microbiology—Functional Microbial Genomics: Academic Press; 2002 p. 241–55.

[24] Rondon MR, Raffel SJ, Goodman RM, Handelsman J. Toward functional genomics in bacteria: analysis of gene expression in *Escherichia coli* from a bacterial artificial chromosome library of *Bacillus cereus*. PNAS USA. 1999;96(11):6451–5. 10339608 10.1073/pnas.96.11.6451 PMC26902

[25] Handelsman J. Metagenomics: application of genomics to uncultured microorganisms. Microbiol Mol Biol Rev. 2004;68(4):669–85. doi: 10.1128/MMBR.68.4.669-685.2004 15590779 10.1128/MMBR.68.4.669-685.2004 PMC539003

[26] Allen HK, Donato J, Wang HH, Cloud-Hansen KA, Davies J, Handelsman J. Call of the wild: antibiotic resistance genes in natural environments. Nat Rev Microbiol. 8(4):251–9. doi: 10.1038/nrmicro2312 20190823 10.1038/nrmicro2312

[27] Kazimierczak KA, Rincon MT, Patterson AJ, Martin JC, Young P, Flint HJ, et al A new tetracycline efflux gene, *tet(40)*, is located in tandem with *tet(O/32/O)* in a human gut firmicute bacterium and in metagenomic library clones. Antimicrob Agents Chemother. 2008;52(11):4001–9. doi: 10.1128/AAC.00308-08 18779355 10.1128/AAC.00308-08 PMC2573101

[28] Riesenfeld CS, Goodman RM, Handelsman J. Uncultured soil bacteria are a reservoir of new antibiotic resistance genes. Environ Microbiol. 2004;6(9):981–9. doi: 10.1111/j.1462-2920.2004.00664.x 15305923 10.1111/j.1462-2920.2004.00664.x

[29] Diaz-Torres ML, Villedieu A, Hunt N, McNab R, Spratt DA, Allan E, et al Determining the antibiotic resistance potential of the indigenous oral microbiota of humans using a metagenomic approach. FEMS Microbiol Let. 2006;258(2):257–62. 16640582 10.1111/j.1574-6968.2006.00221.x

[30] Forsberg KJ, Reyes A, Wang B, Selleck EM, Sommer MO, Dantas G. The shared antibiotic resistome of soil bacteria and human pathogens. Science (New York, NY. 2012;337(6098):1107–11. doi: 10.1126/science.1220761 22936781 10.1126/science.1220761 PMC4070369

[31] Harvey R, Funk J, Wittum TE, Hoet AE. A metagenomic approach for determining prevalence of tetracycline resistance genes in the fecal flora of conventionally raised feedlot steers and feedlot steers raised without antimicrobials. Am J Vet Res. 2009;70(2):198–202. doi: 10.2460/ajvr.70.2.198 19231951 10.2460/ajvr.70.2.198

[32] Seville LA, Patterson AJ, Scott KP, Mullany P, Quail MA, Parkhill J, et al Distribution of tetracycline and erythromycin resistance genes among human oral and fecal metagenomic DNA. Microb Drug Resist. 2009;15(3):159–66. doi: 10.1089/mdr.2009.0916 19728772 10.1089/mdr.2009.0916

[33] Allen HK, Moe LA, Rodbumrer J, Gaarder A, Handelsman J. Functional metagenomics reveals diverse β-lactamases in a remote Alaskan soil. ISME J. 2009;3(2):243–51. doi: 10.1038/ismej.2008.86 18843302 10.1038/ismej.2008.86

[34] Clinical and Laboratory Standards Institute. Performance standards for antimicrobial susceptibility testing; twenty-third informational supplement M100-S22 33 Wayne, PA, USA: Clinical and Laboratory Standards Institute; 2013.

[35] Power P, Radice M, Barberis C, de Mier C, Mollerach M, Maltagliatti M, et al Cefotaxime-hydrolysing β-lactamases in *Morganella morganii*. Eur J Clin Microbiol Infect Dis. 1999;18(10):743–7. 10584905 10.1007/s100960050391

[36] Segel IH. Enzyme kinetics, behavior and analysis of rapid equilibrium and steady-state enzyme systems New York, N.Y.: John Wiley & Sons, Inc.; 1975 210–2.

[37] De Meester F, Joris B, Reckinger G, Bellefroid-Bourguignon C, Frere JM, Waley SG. Automated analysis of enzyme inactivation phenomena. Application to β-lactamases and DD-peptidases. Biochem Pharmacol. 1987;36(14):2393–403. 3038122 10.1016/0006-2952(87)90609-5

[38] Kabsch W. XDS. Acta Crystallogr D Biol Crystallogr. 2010;66:125–32. doi: 10.1107/S0907444909047337 20124692 10.1107/S0907444909047337 PMC2815665

[39] Kabsch W. Integration, scaling, space-group assignment and post-refinement. Acta Crystallogr D Biol Crystallogr. 2010;66:133–44. doi: 10.1107/S0907444909047374 20124693 10.1107/S0907444909047374 PMC2815666

[40] CCP4. The CCP4 suite: programs for protein crystallography. Acta Crystallogr D Biol Crystallogr. 1994;50(Pt 5):760–3. doi: 10.1107/S0907444994003112 15299374 10.1107/S0907444994003112

[41] Murshudov GN, Vagin AA, Dodson EJ. Refinement of macromolecular structures by the maximum-likelihood method. Acta Crystallogr D Biol Crystallogr. 1997;53(Pt 3):240–55. doi: 10.1107/S0907444996012255 15299926 10.1107/S0907444996012255

[42] Painter J, Merritt EA. Optimal description of a protein structure in terms of multiple groups undergoing TLS motion. Acta Crystallogr D Biol Crystallogr. 2006;62(Pt 4):439–50. doi: 10.1107/S0907444906005270 16552146 10.1107/S0907444906005270

[43] Emsley P, Cowtan K. Coot: model-building tools for molecular graphics. Acta Crystallogr D Biol Crystallogr. 2004;60:2126–32. doi: 10.1107/S0907444904019158 15572765 10.1107/S0907444904019158

[44] Schrödinger L. The PyMOL Molecular Graphics System. 1.5.0.4 ed.

[45] Laskowski RA. Enhancing the functional annotation of PDB structures in PDBsum using key figures extracted from the literature. Bioinformatics. 2007;23(14):1824–7. doi: 10.1093/bioinformatics/btm085 17384425 10.1093/bioinformatics/btm085

[46] Oliveira SH, Ferraz FA, Honorato RV, Xavier-Neto J, Sobreira TJ, de Oliveira PS. KVFinder: steered identification of protein cavities as a PyMOL plugin. BMC Bioinformatics. 2014;15:197 doi: 10.1186/1471-2105-15-197 24938294 10.1186/1471-2105-15-197 PMC4071799

[47] Bellais S, Aubert D, Naas T, Nordmann P. Molecular and biochemical heterogeneity of class B carbapenem-hydrolyzing β-lactamases in *Chryseobacterium meningosepticum*. Antimicrob Agents Chemother. 2000;44(7):1878–86. 10858348 10.1128/aac.44.7.1878-1886.2000 PMC89979

[48] Moran-Barrio J, Lisa MN, Larrieux N, Drusin SI, Viale AM, Moreno DM, et al Crystal structure of the metallo-β-lactamase GOB in the periplasmic dizinc form reveals an unusual metal site. Antimicrob Agents Chemother. 2016;60(10):6013–22. doi: 10.1128/AAC.01067-16 27458232 10.1128/AAC.01067-16 PMC5038331

[49] Garcia-Saez I, Mercuri PS, Papamicael C, Kahn R, Frere JM, Galleni M, et al Three-dimensional structure of FEZ-1, a monomeric subclass B3 metallo-β-lactamase from *Fluoribacter gormanii*, in native form and in complex with D-captopril. J Mol Biol. 2003;325(4):651–60. 12507470 10.1016/s0022-2836(02)01271-8

[50] Stoczko M, Frere JM, Rossolini GM, Docquier JD. Postgenomic scan of metallo-β-lactamase homologues in rhizobacteria: identification and characterization of BJP-1, a subclass B3 ortholog from *Bradyrhizobium japonicum*. Antimicrob Agents Chemother. 2006;50(6):1973–81. doi: 10.1128/AAC.01551-05 16723554 10.1128/AAC.01551-05 PMC1479130

[51] Segatore B, Massidda O, Satta G, Setacci D, Amicosante G. High specificity of cphA-encoded metallo-β-lactamase from *Aeromonas hydrophila* AE036 for carbapenems and its contribution to β-lactam resistance. Antimicrob Agents Chemother. 1993;37(6):1324–8. 8328781 10.1128/aac.37.6.1324 PMC187960

[52] Moran-Barrio J, Gonzalez JM, Lisa MN, Costello AL, Peraro MD, Carloni P, et al The metallo-β-lactamase GOB is a mono-Zn(II) enzyme with a novel active site. J Biol Chem. 2007;282(25):18286–93. doi: 10.1074/jbc.M700467200 17403673 10.1074/jbc.M700467200

[53] Mercuri PS, Bouillenne F, Boschi L, Lamotte-Brasseur J, Amicosante G, Devreese B, et al Biochemical characterization of the FEZ-1 metallo-β-lactamase of *Legionella gormanii* ATCC 33297T produced in *Escherichia coli*. Antimicrob Agents Chemother. 2001;45(4):1254–62. doi: 10.1128/AAC.45.4.1254-1262.2001 11257043 10.1128/AAC.45.4.1254-1262.2001 PMC90452

[54] Docquier JD, Pantanella F, Giuliani F, Thaller MC, Amicosante G, Galleni M, et al CAU-1, a subclass B3 metallo-β-lactamase of low substrate affinity encoded by an ortholog present in the *Caulobacter crescentus* chromosome. Antimicrob Agents Chemother. 2002;46(6):1823–30. doi: 10.1128/AAC.46.6.1823-1830.2002 12019096 10.1128/AAC.46.6.1823-1830.2002 PMC127251

[55] Carfi A, Pares S, Duee E, Galleni M, Duez C, Frere JM, et al The 3-D structure of a zinc metallo-β-lactamase from *Bacillus cereus* reveals a new type of protein fold. EMBO J. 1995;14(20):4914–21. 7588620 10.1002/j.1460-2075.1995.tb00174.x PMC394593

[56] Docquier JD, Benvenuti M, Calderone V, Stoczko M, Menciassi N, Rossolini GM, et al High-resolution crystal structure of the subclass B3 metallo-β-lactamase BJP-1: rational basis for substrate specificity and interaction with sulfonamides. Antimicrob Agents Chemother. 2010;54(10):4343–51. doi: 10.1128/AAC.00409-10 20696874 10.1128/AAC.00409-10 PMC2944595

[57] Benvenuti M, Mangani S. Crystallization of soluble proteins in vapor diffusion for X-ray crystallography. Nat Protoc. 2007;2(7):1633–51. doi: 10.1038/nprot.2007.198 17641629 10.1038/nprot.2007.198

[58] Wachino J, Yamaguchi Y, Mori S, Kurosaki H, Arakawa Y, Shibayama K. Structural insights into the subclass B3 metallo-β-lactamase SMB-1 and the mode of inhibition by the common metallo-β-lactamase inhibitor mercaptoacetate. Antimicrob Agents Chemother. 2013;57(1):101–9. doi: 10.1128/AAC.01264-12 23070156 10.1128/AAC.01264-12 PMC3535969

[59] Ullah JH, Walsh TR, Taylor IA, Emery DC, Verma CS, Gamblin SJ, et al The crystal structure of the L1 metallo-β-lactamase from *Stenotrophomonas maltophilia* at 1.7 Å resolution. J Mol Biol. 1998;284(1):125–36. doi: 10.1006/jmbi.1998.2148 9811546 10.1006/jmbi.1998.2148

[60] Lisa MN, Moran-Barrio J, Guindon MF, Vila AJ. Probing the role of Met221 in the unusual metallo-β-lactamase GOB-18. Inorg Chem. 2012;51:12419–25. doi: 10.1021/ic301801h 23113650 10.1021/ic301801h PMC3593996

